# Understanding the Influence of Eating Patterns on Binge Drinking: A Mediation Model

**DOI:** 10.3390/ijerph17249451

**Published:** 2020-12-17

**Authors:** Tamara Escrivá-Martínez, Laura Galiana, Rocío Herrero, Marta Rodríguez-Arias, Rosa Mª Baños

**Affiliations:** 1 Department of Personality, Evaluation and Psychological Treatment, Faculty of Psychology, University of Valencia, 46010 Valencia, Spain; tamaraescrivamartinez@gmail.com; 2 Polibienestar Research Institute, University of Valencia, 46022 Valencia, Spain; ro.herrero.09@gmail.com; 3 Department of Methodology for the Behavioral Sciences, Faculty of Psychology, University of Valencia, 46010 Valencia, Spain; laura.galiana@uv.es; 4 CIBERObn Physiopathology of Obesity and Nutrition, Instituto de Salud Carlos III, 28029 Madrid, Spain; 5 Department of Psychobiology, Faculty of Psychology, University of Valencia, 46010 Valencia, Spain; marta.rodriguez@uv.es

**Keywords:** binge drinking, binge eating, fat intake, youth, undergraduate students

## Abstract

Background: Binge drinking is an important health problem, and it has been related to binge eating and fat intake in animal models, but this relationship has not been tested in humans. The first objective of this study was to analyze whether binge eating and fat intake are related to binge drinking in a youth sample. The second objective was to analyze whether binge eating and fat intake mediate the relationship between individual factors associated with binge eating and fat intake (sex, body mass index (BMI), drive for thinness, body dissatisfaction, eating styles, impulsivity, and food addiction) and binge drinking. Methods: A sample of 428 undergraduate students filled out several questionnaires on binge drinking, binge eating, fat intake, drive for thinness, body dissatisfaction, eating styles, food addiction, and impulsivity. Results: Results showed an excellent model fit: *χ*^2^(25) = 30.342 (*p* = 0.212), comparative fit index (CFI) = 0.992, root mean squared error of approximation (RMSEA) = 0.022 [90% CI = 0.000, 0.047]. Binge eating and fat intake were positively related to binge drinking. Furthermore, emotional eating, external eating, and food addiction showed positive and statistically significant indirect relationships with binge drinking, whereas the relationship with restrained eating was negative. Conclusions: These findings point to the need to use a broader approach in understanding and preventing binge drinking in the youth population by showing the influence of the eating pattern on this problem. This information could be helpful in preventing future behaviors and improving interventions that address health risk behaviors.

## 1. Introduction

The consumption of alcohol by young people has risen sharply in recent decades, and the risk of binge drinking in this population has increasingly been recognized [1]. In Spain, 25% of young people are considered binge drinkers [2]. Commonly, binge drinking is defined as the consumption of five or more drinks in men or four or more drinks in women on a single occasion (about 2 h) [3]. This consumption pattern has become one of the main public health problems because it is associated with multiple adverse consequences, such as low quality of life, unsafe driving, aggressiveness, risky sexual behaviors, cognitive impairment, and emotional and relationship problems [4,5,6,7]. In addition, the high prevalence of binge drinking is especially worrisome in this developmental period, especially in young people, given that they are particularly vulnerable to the neurotoxic effects of alcohol, due to the functional and structural brain changes that occur at this stage [8]. Therefore, it is necessary to identify the risk factors associated with binge drinking in order to understand and prevent this behavior.

Currently, we have a large number of studies in the literature that point to a high comorbidity between eating disorders and alcohol consumption, including empirical studies [9], narrative reviews [10,11], systematic reviews, and meta-analyses [12,13]. However, recently, the emphasis has begun to be placed not only on eating disorders as factors that can help to explain excessive alcohol consumption but also on intake and dietary patterns, such as fat intake and binge eating. In this sense, fat intake is understood as the frequency of consumption of “unhealthy fats”, i.e., saturated fats and trans fats [14], and it has been associated with excessive alcohol consumption [15,16]. Along this line, studies in animal models show a greater preference for fats by rats previously injected with ethanol [15]. In addition, rats that consume more fat show a preference for consuming alcohol over water, a relationship that is not observed in rats that consume carbohydrates [17]. Moreover, evidence in humans also shows higher alcohol consumption by people with high fat consumption [16,18,19]. In this sense, it is hypothesized that this relationship can be explained by the activation of common pathways: fat intake activates the dopaminergic pathway, which is also involved in the processes of reward and motivation of alcohol consumption [20].

In addition, it has been shown that it is not only the type of diet (i.e., frequency of fat intake) but also the type of intake behavior (i.e., binge eating) that seems to contribute to explaining binge drinking [21]. Along this line, binge eating has been defined as the excessive intake of food (fat, carbohydrate, vegetables…) over a short-term period and the lack of control over this intake behavior [22,23]. According to different studies, binge eating and binge drinking share commonalities, regarding the pattern of behavior (impulsivity, loss of control), nature and onset of the problem, and prevalence, among others [24,25,26,27,28]. Given this, some authors explored whether binge eating is related to binge drinking and have concluded that both are associated [9,24,25,29,30,31]. This relation has also been seen in animal models [21].

Regarding binge eating and fat intake, it is well known that there are a variety of individual factors that can underlie high levels of binge eating and fat intake, such as sex, BMI, drive for thinness, body dissatisfaction, eating styles, among others [22,32,33,34,35,36]. Therefore, we aimed to study whether these factors can be indirectly related to binge drinking through their relationship with binge eating and/or fat intake. For example, it is known that people show different tendencies in their food behavior depending on their sex. In this case, literature has shown that there is a difference in prevalence between men and women. While women are more likely to binge [22], men are more likely to have a higher-fat diet in early adulthood [37]. On the other hand, individuals with a higher body mass index (BMI) report a greater number of binges [34] and a higher fat intake [38]. There is also a relationship between personality characteristics and these behaviors. In this sense, impulsivity may lie beneath binge eating and fat intake behaviors. Impulsivity can be defined as a predisposition toward rapid, unplanned reactions to internal or external stimuli without regard to the negative consequences of these reactions [39]. It can be understood that is composed of three components: motor impulsivity (acting without thinking), unplanned impulsivity (a lack of future orientation or forethought), and intentional impulsivity (inability to focus attention or concentrate) [40]. Literature showed that motor and intentional impulsivity seem to underlie both binge eating [41] and unhealthy food consumption [32]. Finally, eating styles may also be at the base of binge eating and fat intake behaviors. Van Strien et al. [42] distinguished three eating styles: emotional eating (eating in response to negative emotions), external eating (eating in response to external cues, such as sight or smell), and restrictive eating (deliberately restricting food to decrease or maintain weight). Studies indicate that both emotional and external eating are positively associated with increased fat intake [35,43], while restrictive eating is associated with decreased fat intake [44]. Furthermore, all three eating styles are associated with binge eating [34], and may act as strong predictors of binge eating [36,45]. Along the same line, the food addiction construct may act as a predictor of the severity of binge eating [33], and those who are addicted to food are also more likely than non-addicts to consume high-fat food [46]. Other variables that predispose to binge eating and fat intake are body dissatisfaction and drive for thinness, as they positively predict binge eating [47] and are negatively associated with fat intake [44,48].

So far, literature has indicated that binge drinking can predict an increase in binge eating and fat intake in young people [24,31]. However, a line of animal research has attempted to study the inverse relationship, suggesting that binge eating and fat intake may also play a role in the development of binge drinking [21]. These studies have explored the relationship, showing that both binge eating and fat intake predict an increase in subsequent ethanol overconsumption in adolescent mice [21]. However, the directionality of this relationship has hardly been studied, and the results are inconclusive [24]. The aforementioned evidence has raised the question of whether binge eating and fat intake might be influencing binge drinking [21], and to our knowledge, no research has focused on evaluating the relation among binge eating, fat intake, and binge drinking in this young population.

Therefore, the first aim of the present study was to explore the relationship between binge eating, fat intake, and binge drinking. Specifically, this model hypothesizes that binge eating and fat intake will positively influence binge drinking. Moreover, taking into account that there are individual factors that may be at the base of binge eating and fat intake, it is hypothesized that those factors can be indirectly related to binge drinking. In this sense, the second objective for this study was to analyze whether binge eating and fat intake mediate the relationship between these individual factors (sex, BMI, drive for thinness, body dissatisfaction, eating styles, impulsivity and food addiction) and binge drinking. With that in mind, we hypothesized that these individual factors may be acting on binge drinking through binge eating and fat intake. To accomplish these objectives, a structural equation model will be tested to study the relationships of binge drinking in the young population.

## 2. Materials and Methods

### 2.1. Participants

The sample of the present study was composed of undergraduate students living in the province of Valencia (Spain). A total of 428 undergraduate students, 324 females (75.7%; mean age 21.04; SD = 4.22) and 104 males (24.3%; mean age 22.27; SD = 5.39), took part voluntarily in the present study. 

### 2.2. Design and Procedure

The study has a cross-sectional design, with data collection carried out at one time point. Data collection followed ethical requirements, and all participants were fully informed about the voluntary nature of their participation and the confidentiality of the collected data. They provided their informed consent prior to being included in the study. Participants were recruited through e-mail, social networks, and word of mouth, and they were directed to a dedicated online survey. The survey was carried out using the Lime Survey web platform, where participants provided demographic data and answered the questionnaires. The study was approved by the Valencia Ethics Committee and performed in accordance with the ethical standards of the 1964 Declaration of Helsinki (Procedure number: H1513854038939).

### 2.3. Measures

The structural model included several exogenous, mediation, and dependent variables. The exogenous variables were:

Sex, with two categories: men and women.

Body mass index (BMI). BMI was calculated by dividing self-reported current weight (in kilograms) by height squared (in meters) [49]. 

Eating disorders, measured with the Eating Disorder Inventory-3 (EDI-3; [50], in its Spanish version [51]). The EDI-3 is a self-report questionnaire consisting of 91 items grouped in 12 subscales designed to assess eating disorder psychopathology and the associated psychological symptoms. In the present study, only the eating disorder risk composite factor was used. The factor consists of 25 items rated on a 6-point Likert scale, and is composed of three scales that measure the risk of having an eating disorder: drive for thinness, bulimia, and body dissatisfaction. The bulimia scale is not used in the present study as it overlapped with the binge eating mediator variable. In this study, Cronbach’s alpha was 0.903 for drive for thinness, and 0.747 for body dissatisfaction. 

Eating styles, using the Dutch Eating Behavior Questionnaire (DEBQ; [42,52]). This questionnaire includes 33 items that measure emotional eating, external eating, and restrained eating. The questionnaire showed adequate levels of internal consistency, with a Cronbach’s alpha value of 0.952 for emotional eating, 0.884 for external eating, and 0.926 for restrained eating.

Impulsivity. This variable was assessed using the Barratt Impulsiveness Scale-15 (BIS-15; [53]; Spanish version: [54]). It is a brief, self-administered scale composed of 15 items grouped in three factors: motor, non-planning, and attentional impulsivity. All the items evaluate impulsivity in different facets. Internal consistency estimates were 0.781 for motor impulsivity, 0.760 for non-planning impulsivity, and 0.690 for attentional impulsivity.

Food addiction, using the modified Yale Food Addiction Scale (mYFAS; [55]). The mYFAS evaluates signs of addictive-like eating behavior. It is composed of nine items consisting of one question from each of the symptom groups that make up the seven diagnostic criteria, plus two individual items that assess the presence of clinically significant impairment and distress. The internal consistency coefficient in this study, measured with Cronbach’s alpha, was 0.769.

As regards the mediation variables, they included:

Binge eating. This variable was assessed using the total score on the Binge Eating Scale (BES; [56]; Spanish version: [57]). The BES is a 16-item self-report questionnaire designed to identify the symptoms associated with binge eating (eating large amounts of food in a short time and feeling loss of control). Internal consistency in this sample was 0.869. 

Fat intake. Fat intake consisted of the total score on the Short Fat Questionnaire (SFQ; [14]). This scale is a 17-item self-report questionnaire that assesses the weekly frequency of fat intake, such as fried food consumption, consumption of sauces or creams, use of fats for cooking, consumption of processed meat, cakes, ice cream, degree of cooking of food, and consumption of milk (with or without fat). For instance: “How many times a week do you eat French fries?” “And chocolate?”, The response scale ranges from never to six times or more. Scores ranged from zero to 62, with a higher score indicating higher frequency of fat intake. This measure has been widely used in the young population to measure fat intake and it has been related to overweight, obesity, and substance use, among others [58,59]. 

This questionnaire had to undergo a rigorous cultural adaptation procedure. It was translated into English by a Spanish-English translator, and subsequently several Spanish reviewers adapted the translated items. The Spanish version of the SFQ is quite similar to the original validation [14]. Internal consistency in this sample, estimated with Cronbach’s alpha, was 0.796.

Finally, the dependent variable in the model was binge drinking. This variable was measured with the main tools to assess binge drinking: item 3 from the AUDIT scale [60] (“How often do you have six or more drinks on one occasion?”), ranging from 0 (never) to 4 (daily or almost daily); and an indicator of binge drinking (“Considering all types of alcoholic beverages, did you ever have five or more drinks (four if you are female) in a two-hour period (one time) in the past month? How many times in the past month?”), ranging from 0 (never) to 4 (4 or more times per week) [61,62]. Internal consistency was 0.735.

### 2.4. Statistical Analyses

Analyses included descriptive statistics and a multiple indicators multiple causes (MIMIC) structural model. The MIMIC model allows us to study the relationships of binge drinking in a context free of measurement error in the main outcome under study, while considering unique relations with specific binge drinking indicators or items. 

**Figure 1:**
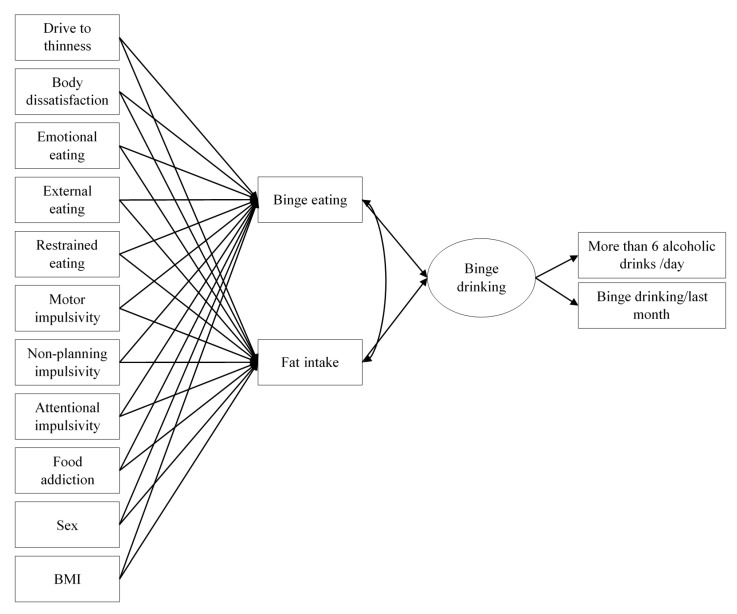
Multiple indicators multiple causes (MIMIC) model to study binge drinking; Notes: For the sake of clarity, standard errors are not shown.

The model hypothesized, estimated, and tested a direct relationship between binge eating (measured with the Binge Eating Scale) and fat intake (measured with the Short Fat Questionnaire) and binge drinking, modeled as a latent factor. In addition, several variables related to binge eating and fat intake were included in the model to test its indirect effects (through binge eating and/or fat intake) on binge drinking. These variables were: sex, BMI, drive for thinness, body dissatisfaction, emotional eating, external eating, restrained eating, motor impulsivity, non-planning impulsivity, attentional impulsivity, and food addiction. The model can be consulted in Figure 1.

The plausibility of the model was assessed using general fit criteria, in addition to examining the analytical fit. Regarding the general model fit, the fit statistics and indices recommended in the literature were used [63,64]: (a) Chi square statistic [65]; (b) comparative fit index (CFI), with values above 0.90 indicating a good representation of the data and, ideally, 0.95; [63]; and (c) root mean squared error of approximation (RMSEA), with values of 0.08 considered a reasonable fit [65], and its 90% confidence interval (CI).

In the case of the analytical fit, factor loadings for the measurement part of the model were examined, along with the effects of the different variables involved. Indirect effects were also calculated, and the CI around the estimate of the effects was also estimated using a bootstrap resampling method. This procedure has been recommended as the best method to generate the required sampling distributions for testing indirect effects [66].

The model was estimated using maximum likelihood with robust standard errors (MLR), using Mplus version 8 [67].

## 3. Results

First, descriptive statistics were calculated for all the variables included in the model. As Table 1 shows, the scores on all the scales were in line with the average in the original validation, showing non-clinical symptomatology for binge eating, food addiction, and impulsivity. Specifically, in relation to the question “How often do you have 6 or more drinks in a single day?”, only 0.7% of the participants reported having had six or more drinks in one day daily or almost daily; 4.0% did this weekly; 22.8%, monthly; 37.8% less than once a month; and 34.6%, never. Regarding the question “Considering all types of alcoholic beverages, did you have five or more drinks (four if you are female) in a two-hour period (one time) in the past month? How many times in the past month?”, only 0.4% of the participants did this twice or three times a week; 9.8%, two to four times a month; 27.4%, once or less than once a month; and 62.4%, never. These values were expected because the sample was drawn from the general population. 

Regarding the study of the relationships of binge drinking, a structural MIMIC model was hypothesized to relate the aforementioned variables to binge drinking. This initial or theoretical model (Figure 1) fitted the data perfectly: *χ*^2^(23) = 28.025 (*p* = 0.214), CFI = 0.992, RMSEA = 0.023 [90% CI = 0.000, 0.048]. The measurement part of the model showed a strong link between the indicators and their corresponding latent variable (binge drinking), with a factor loading of 0.855 (*p* < 0.001) for more than 6 alcoholic drinks/day (item 3 on the AUDIT scale), and 0.663 (*p* < 0.001) for binge drinking episodes in the past month. 

In terms of the relations with binge drinking, both binge eating and fat intake showed a positive and statistically significant relationship. Binge eating showed a positive effect of 0.226 (*p* < 0.001), and fat intake had a positive effect of 0.217 (*p* < 0.001). The correlation between these two indicators was, however, not statistically significant (*r* = −0.021; *p* = 0.684). 

The exogenous variables that were related with binge eating were: drive for thinness (*β* = 0.284, *p* < 0.001), body dissatisfaction (*β* = 0.108, *p* = 0.019), emotional eating (*β* = 0.174, *p* < 0.001), external eating (*β* = 0.119, *p* = 0.004), restrained eating (*β* = −0.122, *p* = 0.026), and food addiction (*β* = 0.443, *p* < 0.001). In the case of fat intake, the statistically significant related variables were: drive for thinness (*β* = −0.184, *p* = 0.018), external eating (*β* = 0.375, *p* < 0.001), restrained eating (*β* = −0.259, *p* < 0.001), motor impulsivity (*β* = 0.132, *p* = 0.013), and food addiction (*β* = 0.177, *p* = 0.008). Correlations among the exogenous variables were in the expected direction and can be consulted in Table 2.

Finally, indirect effects of the exogenous variables on the binge drinking factor were examined. As shown in Table 3, emotional eating, external eating, and food addiction showed positive, statistically significant relations with binge drinking, whereas the relationship with restrained eating was negative.

Overall, 68.3% of binge eating (*R*^2^ = 0.683, *p* < 0.001), 27.0% of fat intake (*R*^2^ = 0.270, *p* < 0.001), and 10.4% of binge drinking (*R*^2^ = 0.104, *p* = 0.015) were explained.

## 4. Discussion

Firstly, the current study examined whether binge eating and fat intake are related to binge drinking in a youth sample. As it was hypothesized, binge eating and fat intake are related to binge drinking, meaning that higher scores on binge eating and higher fat intake behaviors are associated with higher scores on binge drinking behaviors. These findings are in line with recent studies in animal models that showed a causal relationship between a high-fat diet and binge eating behaviors with higher consumption of ethanol [21]. Along the same line, other authors have also proposed a relationship between fat intake and binge drinking [15,16,17,18,19], and between binge eating and binge drinking [24,25]. Furthermore, prospective studies have found that binge eating predicts the risk of frequent drinking and substance abuse [68,69] or drug consumption [13,70]. It has been hypothesized that binge eating induces emotions of guilt and shame, and alcohol and substance use could be used to regulate these negative emotions [71]. 

In addition, there are other mechanisms that may be at the base of binge eating and fat intake, such as eating styles [34,44,45] or food addiction [33,46], among others, that could help explain these relationships.

Taking this into account, secondly, the mediating role of binge eating and fat intake between various individual factors (sex, BMI, drive for thinness, body dissatisfaction, eating styles, impulsivity and food addiction) and binge drinking was examined. Therefore, it was hypothesized that these individual factors may be acting on binge drinking through binge eating and fat intake. As hypothesized, binge drinking is indirectly related to other relevant variables. Specifically, emotional eating, external eating, and food addiction showed positive and statistically significant indirect relationships with binge drinking, whereas the relationship with restrained eating was negative.

First, our results showed that emotional eating was positively related to binge drinking through its relationship with binge eating. These findings replicate some results obtained in previous research. There is evidence that emotional eating can act as a predictor of binge eating [34,36], but it was not yet known whether binge eating could also act as a mediator between emotional eating and binge drinking. In these results, we can conclude that people who eat more in response to negative emotions may be more likely to have higher binge eating scores, and in turn, engaging in binge eating may contribute to higher alcohol consumption.

Second, our results showed that external eating was positively related to drinking behaviors through its relationship with binge eating and fat intake. There is evidence in the literature about the positive relationship between external eating and binge eating [34,45], and between external eating and fat intake [35,43], but our study is the first to analyze the mediating role of binge eating and fat intake in the relationship between external eating and binge drinking. 

Third, our results showed that restrained eating was associated with lower binge drinking behaviors, and this relationship was mediated by the decrease in fat intake. Although the literature has found that restrained eating often leads to higher alcohol consumption, our data could suggest the role of fat intake in better understanding this relationship. That is, people who restrict food and eat less fat will drink less alcohol, possibly because of the calories in both substances. Moreover, a recent study pointed out that alcohol consumption and restrictive behaviors are weakly related [72], and a potential explanatory hypothesis would be that fat intake acts as a mediator between these variables. 

Fourth, food addiction was related to binge eating and fat intake, and through these behaviors, it could lead to an increase in alcohol consumption in the form of binge drinking. Although the literature has pointed out the relationship between food addiction and alcohol [73,74], our study suggests that binge eating and fat intake could act as mediating factors in this relationship.

Several limitations must be highlighted in the present study. The sample was limited to undergraduate students. Therefore, these findings cannot be generalized to other demographic groups such as young people with a low-medium educational status or adults, in whom the prevalence of binge drinking has also been found to be very high. Thus, further studies are required. In the present study, binge drinking was measured with two indicators commonly used in the literature: frequency of consumption of high alcohol doses from the AUDIT-3, and the presence of a binge drinking episode in the past. Both indicators showed an adequate factorial saturation indicating that they worked well as measures of binge drinking, and this result is consistent with the literature [60,62]. Although this is the most common measure used, the research still does not have a standardized measure for binge drinking, and future studies are needed in this field to create a comprehensive and standardized measure. Moreover, other future lines may include testing the model invariance in different subgroups, such as women and men, to detect possible different paths for different populations. Another limitation is the cross-sectional nature of the study, which calls for a cautious interpretation of the results, with other alternative models being possible [75]. Therefore, future research is needed to examine the interactions among these variables in a longitudinal design that makes it possible to assess the temporal relationships between them. In addition, the study was carried out with self-report measures, which may be subject to self-report bias and yield different results compared to clinical interviews or semi-structured assessments [76]. Furthermore, it should also be noted that the explained variance of binge drinking in the present model is small. For future research, it would be necessary to include other relevant factors traditionally related to binge drinking, as well as a sample with a wider variety of characteristics (such as age or the presence of risk factors related to health), to improve the explanatory capability of the current model. Finally, it should be noted the importance for future studies to explore the role of other nutrients, such as carbohydrates, since they can also play an important role in alcohol consumption. In the present study, only the role of high-fat foods has been studied, given its current prevalence of consumption, its negative health consequences, as well as its relationship with alcohol consumption in rodents. This is the first study to explore the relationship between fat intake and binge drinking in young adults, but future research should explore how different nutrients (fats and carbohydrates) separately influence binge drinking.

## 5. Conclusions

This study provides evidence of a direct relationship between binge eating and fat intake and binge drinking, and an indirect relationship between different eating patterns (emotional, restrictive and external eating, and food addiction) and binge drinking, mediated through binge eating and fat intake. The present study is the first to transfer the previous results in mice to humans and assess the influence of eating patterns on binge drinking.

The results of this study can have several implications for health professionals and researchers interested in promoting health and preventing risk behaviors in young people, such as binge drinking, by helping to improve interventions that aim to prevent or decrease binge drinking in young populations. Prevention and intervention strategies could target young people with high scores on these eating patterns to weaken the association between these eating patterns and binge drinking. More specifically, it should be noted that binge eating and fat intake could be a gateway to the initiation and escalation of binge drinking. If we focus on improving these eating patterns in young populations, this can have a dampening effect on the level of alcohol consumption among young people and its consequences. In addition, it should be considered that eating styles (emotional, external, and restrained eating) and food addiction may also precipitate binge drinking through the relationship with binge eating and fat intake. Taking into account this study, it would be appropriate to focus on those young people who have high scores in those eating styles, and who show high scores in food addiction, in order to try to improve those eating patterns as much as possible and thus reduce the binge drinking pattern.

Despite some limitations, these findings provide a unique contribution to the current understanding of the relationship between eating patterns and binge drinking in young people. All these eating patterns are variables to be considered for future interventions and prevention of binge drinking among young people, given their association with binge drinking; however, further research is needed to determine whether these eating patterns can actually explain the high prevalence of binge drinking.

## Figures and Tables

**Table 1:** Descriptive statistics for the variables under study.

Variables	Mean	Standard Deviation
BMI	22.29	3.02
Drive for thinness	7.68	6.94
Body dissatisfaction	11.26	6.50
Emotional eating	25.99	10.70
External eating	30.27	7.33
Restrained eating	21.79	8.70
Motor impulsivity	4.66	4.11
Non-planning impulsivity	8.09	4.41
Attentional impulsivity	7.54	4.65
Food addiction	5.69	4.65
Binge Eating Scale	7.80	6.63
Short Fat Questionnaire	22.47	7.92
More than 6 alcoholic drinks/day	0.99	0.89
Binge drinking/past month	0.48	0.68

**Table 2:** Correlations among the exogenous variables in the MIMIC model.

Variables	1	2	3	4	5	6	7	8	9	10
1. Drive for thinness	--									
2. Body dissatisfaction	0.608 ***	--								
3. Emotional eating	0.377 ***	0.383 ***	--							
4. External eating	0.175 **	0.177 **	0.497 ***	--						
5. Restrained eating	0.797 ***	0.504 ***	0.405 ***	0.226 ***	--					
6. Motor impulsivity	0.169 **	0.197 **	0.138 *	0.163 **	0.190 **	--				
7. Non-planning impulsivity	0.001 n.s.	0.071 n.s.	0.104 n.s.	0.103 n.s.	0.020 n.s.	0.327 ***	--			
8. Attentional impulsivity	0.132 *	0.264 ***	0.230 ***	0.214 ***	0.101 n.s.	0.284 ***	0.266 ***	--		
9. Food addiction	0.558 ***	0.512 ***	0.613 ***	0.403 ***	0.561 ***	0.299 ***	0.092 n.s.	0.225 ***	--	
10. Sex	0.247 ***	0.147 **	0.210 ***	0.143 **	0.213 ***	−0.093 n.s.	−0.117 *	−0.107 *	0.062 n.s.	--
11. BMI	0.260 ***	0.317 ***	0.159 **	−0.018 n.s.	0.242 ***	0.062 n.s.	0.086 n.s.	0.105 *	0.184 **	−0.220 ***

Notes: Sex was coded as: 0 = men, 1 = women; positive correlations indicate higher levels for women, whereas negative correlations indicate higher levels for men; n.s. *p* > 0.050; * *p* < 0.050; ** *p* < 0.010; *** *p* < 0.001.

**Table 3:** Indirect effects of the exogenous variables on the binge drinking factor in the MIMIC model.

Variables	Effect through Binge Eating [95% CI]	Effect through Fat Intake [95% CI]	Total Effect [95% CI]
Drive for thinness	0.064 ** [0.029, 0.120]	−0.040 * [−0.096, −0.008]	0.024 n.s. [−0.031, 0.085]
Body dissatisfaction	0.024 * [0.005, 0.054]	0.006 n.s. [−0.019, 0.036]	0.031 n.s. [−0.004, 0.076]
Emotional eating	0.039 ** [0.016, 0.076]	0.002 n.s. [−0.025, 0.032]	0.042 * [0.006, 0.089]
External eating	0.027 * [0.009, 0.057]	0.081 ** [0.035, 0.143]	0.108 *** [0.055, 0.175]
Restrained eating	−0.028 n.s. [−0.064, 0.005]	−0.056 ** [−0.117, −0.020]	−0.084 ** [−0.151, −0.038]
Motor impulsivity	−0.011 n.s. [−0.037, 0.007]	0.029 * [0.006, 0.068]	0.017 n.s. [−0.013, −0.057]
Non-planning impulsivity	0.019 n.s. [−0.003, 0.042]	−0.017 n.s. [−0.047, 0.001]	0.002 n.s. [−0.028, 0.029]
Attentional impulsivity	0.004 n.s. [−0.011, 0.023]	−0.006 n.s. [−0.034, 0.015]	−0.002 n.s. [−0.033, 0.026]
Food addiction	0.100 *** [0.045, 0.166]	0.038 * [0.010, 0.087]	0.139 *** [0.072, 0.218]
Sex	0.005 n.s. [−0.008, 0.023]	−0.004 n.s. [−0.029, 0.017]	0.002 n.s. [−0.026, 0.027]
BMI	0.013 n.s. [0.000, 0.032]	−0.011 n.s. [−0.036, 0.007]	0.002 n.s. [−0.024, 0.028]

Notes: Sex was coded as: 0 = men, 1 = women; positive correlations indicate higher levels for women, whereas negative correlations indicate higher levels for men; n.s. *p* > 0.050; * *p* < 0.050; ** *p* < 0.010; *** *p* < 0.001.

## Abbreviations

CFI	Comparative Fit Index
CI	Confidence Interval
MIMIC	Multiple Indicators Multiple Causes
MLR	Maximum Likelihood with Robust standard errors
RMSEA	Root Mean Squared Error of Approximation
